# Stochastic resonance in MoS_2_ photodetector

**DOI:** 10.1038/s41467-020-18195-0

**Published:** 2020-09-02

**Authors:** Akhil Dodda, Aaryan Oberoi, Amritanand Sebastian, Tanushree H. Choudhury, Joan M. Redwing, Saptarshi Das

**Affiliations:** 1 Department of Engineering Science and Mechanics, Pennsylvania State University, University Park, PA 16802 USA; 2 2D Crystal Consortium-Materials Innovation Platform (2DCC-MIP), Pennsylvania State University, University Park, PA 16802 USA; 3 Department of Materials Science and Engineering, Pennsylvania State University, University Park, PA 16802 USA; 4 Materials Research Institute, Pennsylvania State University, University Park, PA 16802 USA

**Keywords:** Two-dimensional materials, Optical sensors

## Abstract

In this article, we adopt a radical approach for next generation ultra-low-power sensor design by embracing the evolutionary success of animals with extraordinary sensory information processing capabilities that allow them to survive in extreme and resource constrained environments. Stochastic resonance (SR) is one of those astounding phenomena, where noise, which is considered detrimental for electronic circuits and communication systems, plays a constructive role in the detection of weak signals. Here, we show SR in a photodetector based on monolayer MoS_2_ for detecting ultra-low-intensity subthreshold optical signals from a distant light emitting diode (LED). We demonstrate that weak periodic LED signals, which are otherwise undetectable, can be detected by a MoS_2_ photodetector in the presence of a finite and optimum amount of white Gaussian noise at a frugal energy expenditure of few tens of nano-Joules. The concept of SR is generic in nature and can be extended beyond photodetector to any other sensors.

## Abstract (web summary)

Here, the authors take advantage of stochastic resonance in a photodetector based on monolayer MoS_2_ for measuring otherwise undetectable, ultra-low-intensity, subthreshold optical signals from a distant light emitting diode in the presence of a finite and optimum amount of white Gaussian noise.

## Introduction

Stochastic resonance (SR) is a remarkable phenomenon that shows the constructive role of noise in information processing contrary to the conventional wisdom that noise is a nuisance. In SR, a sensory system embedded in a noisy environment can detect weak time-variant signals when the noise intensity reaches some finite and appropriate level. Over the last three decades, SR has been discovered and demonstrated in a wide variety of geological, biological, and physical systems on almost every possible scale, highlighting its importance in both natural and artificial designs^1–12^. The concept of SR was first proposed in the context of a periodic recurrence of the Earth’s ice ages^3^ and was subsequently taken up and applied in sensory neurobiology for explaining animal behaviors leading to evolutionary success. For example, the electroreceptors located in the rostrum of paddlefish use SR to detect their feed zooplankton *Daphnia*, which predominantly dwell near river beds, where poor light and turbid water limit vision^5^. In behavioral experiments, it was shown that with optimal amount of noise a paddlefish is able to capture more distant plankton than without noise, whereas excessive noise led to almost no feeding. A similar behavioral SR was observed in the escape response of crickets from a predator wasp^6^. At the cellular level, SR is observed in the mechanoreceptor neurons located in the tail fans of crayfish when avoiding an approaching predator^4^ and at the most fundamental molecular level, SR plays a critical role in regulating the signal transduction by ion channels^13^. SR has already found application in medical care to restore the sensitivity of dysfunctional organs responsible for hearing^14^, tactile^15^ or visual sensations^16^ or for the balance control^17^ by means of applying the right amount of noise. The first demonstration of SR in electronic devices was based on a noise-driven Schmitt trigger circuit^10^. SR has now been observed in a wide variety of physical systems including bi-stable ring lasers^9^, optical heterodyning^18^, electronic paramagnetic resonance^19^, superconducting quantum interference devices (SQUID)^7^, and tunnel diodes^8^, among others. While there are some reports of experimental demonstration of SR in field-effect transistors (FETs) based on carbon nanotubes^20–22^, GaAs nanowires^23–26^, and organic semiconductors^27^, the strength of SR is yet to be exploited in solid-state sensors.

While some areas of science, especially sensory neurobiology, have invested significantly in research related to SR, sadly SR has remained largely unrecognized by the device community. This is not entirely surprising since the conventional approaches for improving the detection limit of various solid-state sensors continue to be extremely successful, inhibiting any radical ideas like SR from flourishing. There is no dispute that lock-in amplifiers, oscillators, low noise amplifiers, etc., will persist to advance and remain relevant and essential for electronic system design. However, these traditional approaches remain power hungry, area inefficient, and resource extensive. Fortunately, new technologies invoke new challenges, which foster new scientific discoveries and often rekindle older concepts that have been either abandoned or overlooked. Such is the case for SR with the emergence of the era of Internet of Things (IoT), one of the fastest-growing technologies which will rely on ~200 billion smart sensors by the end of the year 2020. These devices will bridge the physical world with the world of computing to revolutionize all aspects of human life, including healthcare, entertainment, home automation, wearables, telemetry, security, infrastructure, agriculture, and so on and so forth. However, the biggest challenges towards the realization of this IoT vision is the energy, and resource efficiency of these tens of billions of sensors. Most of these devices will be deployed at remote, inaccessible, and resource-constrained locations, where unlimited electrical energy is unavailable. While SR based sensors are unlikely to replace all conventional sensors, the energy benefits of SR can no longer be ignored in the context of rapidly evolving IoT technology.

In this article, we exploit SR for detecting ultra-low-intensity and otherwise undetectable optical signals from a distant light-emitting diode (LED) using a monolayer MoS_2_ based photodetector with unprecedented energy efficiency. We demonstrate that the detection of subthreshold LED illumination is possible by adding a finite and optimum amount of white Gaussian noise. The noise can be either in the source of the weak periodic signal or externally added through an on-chip noise generator. We also show that the concept of SR can be exploited to extend the detection limit of state-of-the-art commercial photodetectors such as Si photodiodes. Finally, we show that subthreshold signal detection can be achieved at the frugal energy expenditure of few nano-Joules by the MoS_2_ photodetector and a few micro-Joules by the Si photodiode, emphasizing the energy benefits of SR. While our exhibition of SR primarily involves photodetectors, the concept of SR is generic in nature and can be extended to any sensors. SR-based sensors can mark a paradigm shift from conventional approaches by harnessing the constructive role of noise in the detection of subthreshold signals.

## Results

### Monolayer MoS_2_ synthesis, characterization, and device fabrication

MoS_2_ is a layered two-dimensional (2D) semiconductor that belongs to the family of transition metal dichalcogenides (TMDCs) with the general formula of MX_2_, where M represents the transition metal (Mo, W, etc.) and X represents chalcogen (S, Se, Te, etc.)^28–30^. These materials exhibit strong intra-layer covalent bonding and weak van der Waals (vdW) interlayer coupling that allows separation of monolayers from their respective bulk crystal with unprecedented electronic and optoelectronic properties, making them attractive for the next generations of advanced sensors. For example, monolayer TMDCs are direct bandgap semiconductors, fostering enhanced light-matter interaction and thereby enabling photonic devices^31^. Furthermore, the atomically thin body nature of TMDCs allows for superior electrostatic control of charge carriers, which is critical for achieving low power operation^32^. While most of the preliminary device studies are performed on micromechanically exfoliated single-crystal flakes, transitioning to technology requires manufacturable solutions. Therefore, the present study uses large-area growth of monolayer MoS_2_ film using a scalable bottom-up synthesis technique, namely the metal-organic chemical vapor deposition (MOCVD), on a sapphire substrate at 1000 °C. Higher growth temperatures, hydride chalcogen precursors, and epitaxial growth offer better crystallinity^33^, reduced carbon contamination^34^, and hence higher device performance in terms of ON current and carrier mobility, as we shall demonstrate and discuss later.

**Fig. 1:**
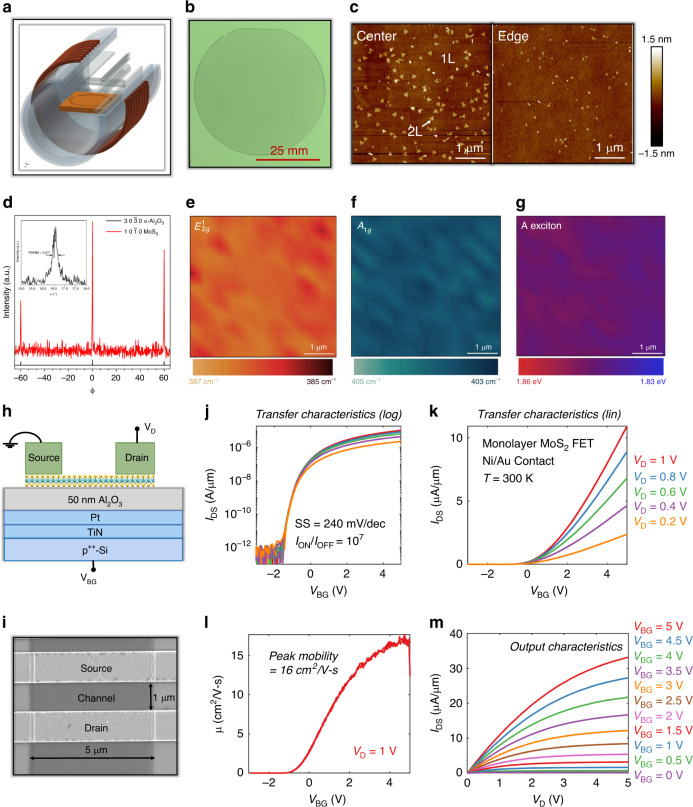
Monolayer MoS_2_ synthesis, characterization, and device fabrication. **a** Schematic of cold-wall horizontal reactor setup. **b** Coalesced monolayer MoS_2_ film grown on a 2-inch sapphire substrate using MOCVD at 1000 °C with Mo(CO)_6_ and H_2_S as precursors. **c** AFM images at the center and edge of the wafer showing uniform surface coverage, film morphology, and thickness. **d** In-plane X-ray diffraction showing the epitaxial relation between MoS_2_ and sapphire as (10$\bar 1$0) MoS_2_ ‖ (10$\bar 1$0) α-Al_2_O_3_. The inset shows a narrow full-width half-maximum (FWHM) of 0.27°, which emphasizes the high crystalline quality of these monolayer films. Raman map of (**e**) ${\mathrm{E}}_{2{{g}}}^1$ and (**f**) A_1g_ peaks show <5% variation and the peak separation of 18 cm^−1^ confirms monolayer MoS_2_. **g** Photoluminescence (PL) map shows an intense peak at 1.84 eV, which is attributed to the indirect to direct bandgap transition in monolayers and is severely suppressed in multilayers of MoS_2_. **h** Schematic and **i** SEM image of monolayer MoS_2_ FET. **j** Logarithmic scale and **k** linear scale transfer characteristics of monolayer MoS_2_ FET measured at different drain biases (*V*_*D*_). **l** Mobility plot and **m** output characteristics measured at different back gate biases (*V*_BG_).

A schematic of the cold-wall horizontal reactor setup is shown in Fig. 1a. Molybdenum hexacarbonyl, Mo(CO)_6_, and H_2_S were introduced into the growth zone in a H_2_ carrier gas at 1000 °C for 18 min to obtain a coalesced monolayer MoS_2_ film on a 2-inch sapphire substrate as shown in Fig. 1b. Surface coverage, film morphology, and thickness were measured using an atomic force microscope (AFM) at the center and edge of the wafer as shown in Fig. 1c. As is evident, the bilayer density was found to be higher at the center than the edges. The crystalline quality was assessed using in-plane X-ray diffraction^35^. The φ-scan in Fig. 1d establishes the epitaxial relation between MoS_2_ and sapphire as (10$\bar 1$0) MoS_2_ ‖ (10$\bar 1$0) α-Al_2_O_3_. In addition, the inset shows a narrow full-width half-maximum (FWHM) of 0.27°, which emphasizes the high crystalline quality of these monolayer films. Following the synthesis, the MoS_2_ film was transferred from the growth substrate to the device fabrication substrate using the PMMA-assisted wet transfer process^36^. We have used atomic layer deposition (ALD) grown 50 nm Al_2_O_3_ (*ε*_ox_ ~ 9) on Pt/TiN/p^++^-Si for the device fabrication. The transferred film quality was assessed using Raman spectroscopy and photoluminescence (PL) maps, as shown in Fig. 1e–g. The $E_{2g}^1$ (Fig. 1e) and A_1*g*_ (Fig. 1f) peak positions vary <5% over the entire map and the peak separation of 18 cm^−1^ corresponds to monolayer MoS_2_^37^. The uniform and intense PL peak observed at 1.84 eV (Fig. 1g) is attributed to the indirect to direct bandgap transition at the K point in the Brillouin zone in monolayers and is severely suppressed in multilayers of MoS_2_^38,39^.

After ensuring the high quality and uniformity of the film, we fabricated monolayer MoS_2_ FET based photodetectors as shown using the schematic and scanning electron microscope (SEM) image in Fig. 1h and i, respectively. The devices have 1 μm channel length, 5 μm channel width, 40 nm Ni/ 30 nm Au as the source/drain contacts, and 50 nm Al_2_O_3_ as the back-gate dielectric. The use of thin and high-k dielectric material such as Al_2_O_3_ compared to conventional 300 nm of SiO_2_ allows supply voltage scaling due to better electrostatic gate control and aids the ultra-low-power operation of the photodetector. Further details on the synthesis, film transfer, characterization, device fabrication, and electrical measurements of monolayer MoS_2_ photodetector are provided in the “Method” section. Figure 1j and k show the transfer characteristics, i.e. source to drain current (*I*_DS_) as a function of the back gate voltage (*V*_BG_) for the monolayer MoS_2_ FET measured at different drain biases (*V*_*D*_) in the logarithmic and linear scales, respectively. The MoS_2_ FET exhibits unipolar n-type transport owing to the phenomenon of metal Fermi level pinning close to the conduction band facilitating electron injection, which is consistent with all other previous reports^40,41^. The device shows excellent ON/OFF current ratio in excess of 10^7^, subthreshold slope (SS) of less than 294 mV/decade, none to minimal drain induced barrier lowering (DIBL)^42^, absence of any gate hysteresis even under ambient measurements, low gate leakage, and low contact resistance, thus indicating high quality of the gate dielectric, semiconducting MoS_2_ channel, and dielectric and contact interfaces (see Supplementary Note 1). Combined, the device exhibits low voltage operation under 5 V for both the back-gate bias and drain bias. The threshold voltage (*V*_TH_) was found to be ~0.2 V and the field-effect mobility (*μ*_FE_) value was found to be ~16 cm^2^ V^−1^ s^−1^ extracted from the peak transconductance, as shown in Fig. 1l. The *μ*_FE_ value is comparable to exfoliated single-crystal monolayer MoS_2_. This is also reflected in the output characteristics (i.e., *I*_DS_ versus *V*_*D*_ for different *V*_BG_) of the MoS_2_ FET, with the ON current reaching as high as ~35 μA/μm at *V*_*D*_ = 5 V for an inversion charge carrier density of ~5 × 10^12^ cm^−2^, as shown in Fig. 1m. We also measured the transfer characteristics of 10 devices across the entire substrate and found consistent device performance, confirming uniform wafer-scale growth of high-quality monolayer MoS_2_.

### Demonstration of SR in monolayer MoS_2_ FET

**Fig. 2:**
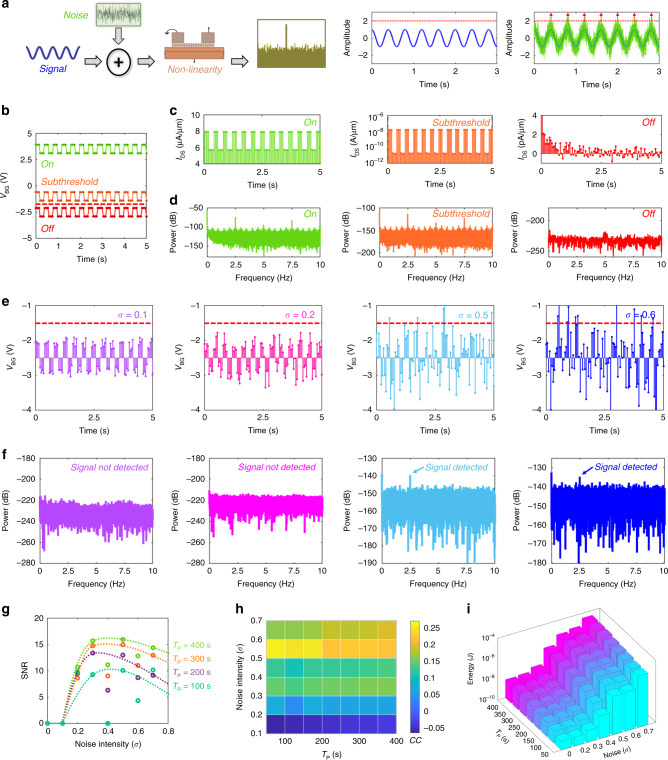
Demonstration of stochastic resonance (SR) in monolayer MoS_2_ field-effect transistor (FET). **a** Schematic showing the basic concept of SR with three essential components: a nonlinear thresholding device, a weak coherent input such as a periodic signal, and a source of noise. When the noise intensity reaches some finite and appropriate level, the system can detect the weak time-variant signal, which otherwise lies below the detection threshold of the sensor. **b** A 2.5 Hz square wave of amplitude 0.4 V is applied to the back-gate of the monolayer MoS_2_ FET at different operating regimes: ON-state (*V*_BG_ = 3.5 V), subthreshold (*V*_BG_ = −1 V), and OFF-state (*V*_BG_ = −2.5 V). **c** Output current (*I*_DS_) and **d** corresponding PSD plots obtained using the FFT. Current sampling was done at 20 Hz for ~100 s. In the ON-state and subthreshold regime the signal is detected, whereas, in the OFF-state, *I*_DS_ is obscured by the noise floor and corresponding peaks are absent in the PSD. **e** In order to detect the signal in the OFF-state, Gaussian noise of different standard deviations (σ) are superimposed on the square wave centered at *V*_BG_ = −2.5 V. **f** PSD of measured *I*_DS_. The current sampling was done at 20 Hz for ~400 s. The signal is detected for an optimum amount of Gaussian noise, confirming SR in MoS_2_ FET. **g** SNR as a function of σ for various total sampling time (*T*_P_). **h** Color map of the correlation coefficient (CC) between *I*_DS_ and *V*_BG_. **i** Energy consumption as a function of σ for various *T*_P_. Clearly, optimum signal detection can be achieved with energy as frugal as 10–100 nJ corresponding to σ = 0.5 V.

Figure 2a explains the basic concept of SR that requires three essential components: (i) a nonlinear thresholding device; (ii) a weak coherent input (such as a periodic signal) which is below the detection threshold of the device; and (iii) a source of noise either inherent to the device or added externally to the coherent input. Before moving on to SR in MoS_2_ photodetector, first, we illustrate the concept of SR using a monolayer MoS_2_ FET. Figure 2b shows a 2.5 Hz periodic input signal of amplitude 0.4 V applied to the back gate at different operating regimes: ON-state (*V*_BG_ = 3.5 V), subthreshold (*V*_BG_ = −1 V), and OFF-state (*V*_BG_ = −2.5 V). Figure 2c and d, respectively, show *I*_DS_ and the corresponding power spectral density (PSD) obtained using fast Fourier transforms (FFTs). The current sampling was done at 20 Hz for ~100 s. As expected, *I*_DS_ follows the *V*_BG_ waveform in the ON state owing to linear device operation and the corresponding PSD shows a sharp peak at 2.5 Hz, which is the input signal frequency. In the subthreshold regime, the output current waveform is distorted since the device characteristics exhibit nonlinearity owing to the exponential dependence of *I*_DS_ on *V*_BG_. The corresponding PSD of *I*_DS_, however, detects the input signal at 2.5 Hz. Finally, in the OFF state (*V*_BG_ < −1.5 V), *I*_DS_ is obscured by the noise floor and correspondingly peaks are absent in the PSD.

In order to detect the periodic signal in the OFF state, Gaussian noise with different values of standard deviation (*σ*) was added to the periodic input signal (*V*_BG_), as shown in Fig. 2e. Figure 2f shows the corresponding PSD of *I*_DS_. Current sampling was done at 20 Hz for ~400 s. Remarkably, in the presence of an optimum amount of Gaussian noise, the PSD data exhibits a peak at the input signal frequency of 2.5 Hz. Figure 2g shows the SNR as a function of σ for various total sampling time (*T*_P_). The histograms of *V*_BG_ distribution for the bi-level square wave input signal shown in the Supplementary Note 2 help in explaining the nonmonotonic trend in the SNR. For very low variance Gaussian noise (*σ* < 0.2 V), none of the two signal levels crosses the detection threshold (*V*_BG_ = −1.5 V), whereas, for very high variance Gaussian noise (*σ* > 0.6 V), both signal levels cross the detection threshold and obscures the periodicity of the signal. However, for appropriate noise variance, mostly one of the two signal levels cross the detection threshold allowing the signal frequency to be detected and as such the PSD exhibits a peak at 2.5 Hz. Note that the SNR can be increased by increasing the *T*_P_ since it allows the subthreshold signal to cross the detection threshold a greater number of times. A similar observation is made in Fig. 2h showing the colormap of the correlation coefficient (CC) between *I*_DS_ and *V*_BG_. Finally, Fig. 2i shows the energy consumption by the MoS_2_ FET for detecting the weak period signal as a function of σ for different *T*_P_ calculated based on *E* = *I*_DS_*V*_*D*_*T*_*p*_. Clearly, optimum signal detection can be achieved with energy as frugal as 10–100 nJ corresponding to *σ* = 0.5 V.

To elucidate some key aspects of SR, we have used the Virtual Source (VS) model^43–45^, described in Supplementary Note 3, for fitting the monolayer MoS_2_ FET characteristics. In the VS model, both the subthreshold and the above threshold FET characteristics are captured through a single semi-empirical and phenomenological relationship that describes the transition in channel charge from weak to strong inversion. The model parameters were obtained from experimentally measured device characteristics. First, we show that while noise with optimum standard deviation yields maximum enhancement in the SNR, noise with any finite non-zero standard deviation can achieve the same SNR when averaged over longer period. This is because the tail of any Gaussian distribution with finite standard deviation extends to infinity. Therefore, there is always a finite probability that the on-signal will cross the threshold even when the standard deviation of noise is small. By sampling the signal over a longer time, one ensures enough threshold crossing events at the signal frequency, which then adds up in the PSD to enhance the SNR. Supplementary Note 4 shows the simulated PSD of *I*_DS_ in response to a 2.5 Hz periodic input signal of amplitude 0.2 V with Gaussian noise of different standard deviations (*σ*) applied to the back gate in the OFF-state (*V*_BG_ = −2.5 V) for an averaging period that ranges from 10^3^ s to 10^7^ s and the corresponding SNR values. As expected, no peaks are observed for *σ* = 0 V, i.e., without noise even if the sampling is done for an infinitely long period of time. For small *σ* (*σ *= 0.2 V), the peak appears after sampling for 10^7^ s, whereas, for optimum *σ*  (*σ *= 0.5 V), the peak appears after 10^3^ s. These simulation results indicate that the SR induced enhancement of SNR can be achieved using any finite value of *σ* at the expense of increased latency. Finally, Supplementary Note 5 shows that the signal frequency does not impose any limitation on the exploitation of SR for the detection of weak periodic signals. In fact, an earlier work has explored THz wave detection by GaAs nanowire-based FETs using SR^46^.

### Detection limit for monolayer MoS_2_ photodetector

**Fig. 3:**
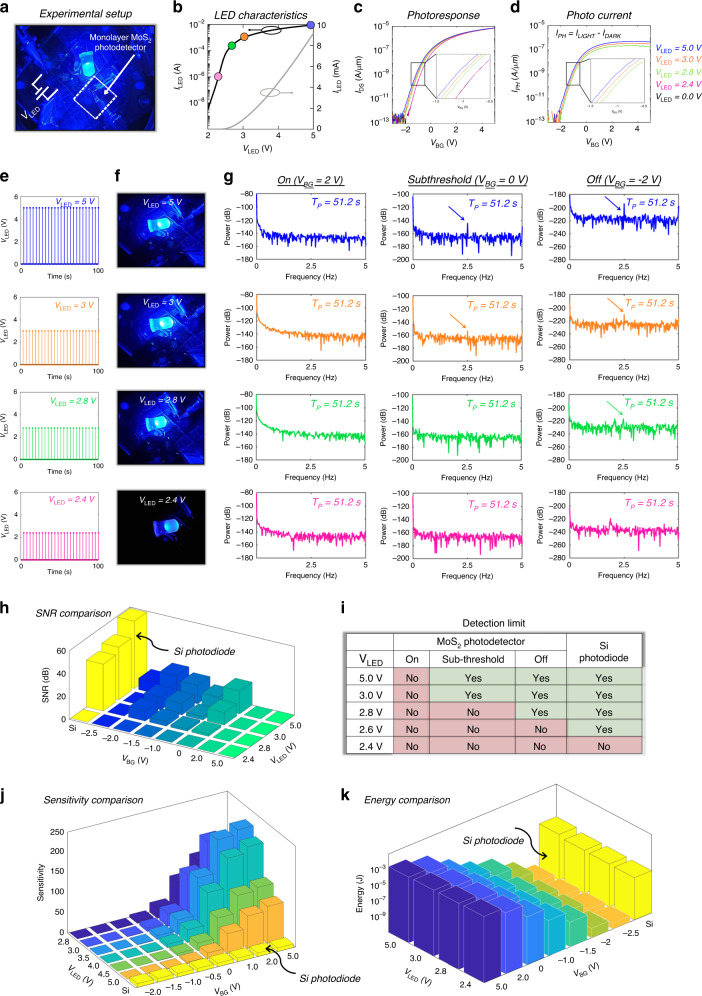
Detection limit of monolayer MoS_2_ photodetector. **a** Experimental setup showing the photodetector chip based on monolayer MoS_2_ FET placed at a distance of ~1 cm from a blue LED. **b** Current (*I*_LED_) versus voltage (*V*_LED_) characteristics of the LED. **c** Transfer characteristics of the MoS_2_ FET under dark and illumination corresponding to different *V*_LEG_. **d** The extracted photocurrents (*I*_PH_). **e** 2.5 Hz periodic signals of different amplitudes applied to the LED and **f** corresponding snapshots of the glowing LED. The LED intensity drops linearly for 5 V > *V*_LED_ > 2.8 V and exponentially for *V*_LED_ < 2.8 V in accordance with the output characteristics of the LED, as indicated using the colored circles in (**b**). **g** PSD plots obtained from FFT of *I*_DS_ for a total duration of *T*_P_ = 51.2 s in different operation regimes in response to periodic LED signals in (**e**). Peaks observed in the PSD at 2.5 Hz are indications of successful detection of the LED signal. **h** Extracted SNR in different detection regimes for various LED illuminations. For comparison, we have also included the results obtained from the same experiments performed on a commercial Si photodiode in Supplementary Note 6*.* **i** Table summarizing the detection limit for the blue light by MoS_2_ photodetector and Si photodiode. **j** Sensitivity of the MoS_2_ photodetector in different detection regimes. The sensitivity (*S*) of a photodetector is defined as the ratio of photocurrent (*I*_PH_) to the luminous flux (*L*_*ϕ*_). The rated sensitivity of the Si photodiode (*S* = 9 nA/lx) and its photoresponse to different *V*_LED_ were used for calibrating *L*_*ϕ*_, which were subsequently used to calculate the sensitivity of MoS_2_ photodetector. **k** Comparison of energy expenditure for the Si photodiode and MoS_2_ photodetector for detection of above threshold signals. The MoS_2_ photodetector offers orders of magnitude higher energy efficiency when operated in the subthreshold regime, owing to significantly lower dark current.

Figure 3a shows the experimental set-up used for the demonstration of SR, consisting of a monolayer MoS_2_ photodetector and a blue LED spaced  ~1 cm away from the photodetector. Note that monolayer MoS_2_ is a direct bandgap (*E*_G_ = 1.84 eV) semiconductor with high quantum yield^30^, which makes it attractive for photonic device applications^47–49^. While most of the studies reporting the optical response of monolayer MoS_2_ are based on LASER excitation, we have used an LED source to mimic ultra-low-intensity realistic light sources. Figure 3b shows the current (*I*_LED_) versus voltage (*V*_LED_) characteristics of the LED. Figure 3c and d, respectively, show the output current (*I*_DS_) and extracted photocurrent (*I*_PH_) in the MoS_2_ photodetector under different LED illuminations. In order to determine the sensitivity and detection threshold for the MoS_2_ photodetector, we applied 2.5 Hz periodic LED signals of different amplitudes, as shown in Fig. 3e. The snapshots of the glowing LED at the corresponding *V*_LED_ biases are shown in Fig. 3f. The LED intensity drops linearly for 5 V > *V*_LED_ > 2.8 V and exponentially for *V*_LED_ < 2.8 V in accordance with the output characteristics of the LED, as indicated using the colored circles in Fig. 3b. Figure 3g shows the corresponding PSD plots obtained from the FFT of the output current (*I*_DS_) measured in the monolayer MoS_2_ photodetector for a total duration of *T*_P_ = 51.2 s in the ON-state (*V*_BG_ = 2 V), subthreshold (*V*_BG_ = 0 V), and OFF-state (*V*_BG_ = −2 V). In all cases, current sampling was done at 10 Hz. Peaks observed in the PSD at 2.5 Hz are indications of successful detection of the periodic LED signal by the MoS_2_ photodetector. Figure 3h shows the extracted SNR in different detection regimes for various LED illuminations and Fig. 3i summarizes the detection limit for the blue light by the MoS_2_ photodetector. For comparison, we have also included the results obtained from the same experiments performed on a commercial Si photodiode in Supplementary Note 6.

As expected, the SNR decreases monotonically with the decreasing LED signal intensity for both the Si photodiode and the MoS_2_ photodetector, irrespective of the biasing regime for the latter. However, a closer look reveals a non-monotonic trend in the SNR for the MoS_2_ photodetector as a function of *V*_BG_ for any given LED intensity. This can be explained using the phototransduction mechanism and current transport in monolayer MoS_2_. Under the illumination of the blue LED (450–500 nm), electron-hole pairs are generated in the MoS_2_ channel since the incident photon energy exceeds ∼1.84 eV, the optical band gap of the monolayer MoS_2_. The photoexcited carriers constitute the photocurrent that adds to the channel dark current due to drift under the applied drain bias. In the ON-state, the device dark current obscures the photocurrent generated in response to even the brightest LED signal, i.e., *V*_LED_ = 5 V, limiting signal detection and reducing the SNR to zero. By operating the device in the subthreshold, the dark current can be reduced exponentially, allowing the detection of low-intensity LED signals, e.g. down to *V*_LED_ = 3 V. However, as *V*_LED_ is further reduced, the light intensity from the LED drops exponentially and so does the photocurrent generated in the device, making it difficult to detect the LED signal even in the subthreshold regime. We found that the detection threshold of the MoS_2_ photodetector can be extended to *V*_LED_ = 2.6 V by operating the device in the OFF-state. However, for *V*_LED_ < 2.6 V, the LED signal is beyond the detection limit irrespective of the operating regime of the MoS_2_ photodetector.

For a given LED intensity, it is expected that the photocurrent generation in monolayer MoS_2_ should be constant irrespective of the applied gate bias, as is found in the case of Si photodiode. However, that is clearly not the case according to Fig. 3d, which shows a monotonic decrease in the photocurrent with negative gate biases. This can be explained using carrier trapping at the interface between Al_2_O_3_ and MoS_2_ similar to the SiO_2_ and MoS_2_ interface as reported in the literature^50^. These interface trap states originate from the dangling Al−O bonds at the surface. Photocarriers trapped in these donor-like interface states follow a Fermi−Dirac distribution, with the Fermi level being determined by the equilibrium Fermi level of the MoS_2_ channel^51–53^. For larger negative *V*_BG_, more trap states become available for carrier capture, leading to a decrease in photocurrent and SNR. Supplementary Note 7 shows the sweep rate-dependent hysteresis in MoS_2_ FET in dark and under illumination, which highlights the trap-assisted phototransduction mechanism in the subthreshold regime. We also investigated light-enhanced gate leakage in Supplementary Note 8 and found no significant contribution due to the same in the phototransduction mechanism in the monolayer MoS_2_ photodetector. Nevertheless, the above experiments established *V*_LED_ > 2.6 V as a detection limit for the MoS_2_ photodetector.

In comparison, the detection threshold for the Si photodiode was found to be *V*_LED_ > 2.4 V, below which the reverse dark current dominates, limiting the detection of low-intensity blue light from the distant LED. The rated reverse dark current for the Si photodiode was found to be 2 nA (typical), and 30 nA (maximum). The Si photodiode used in our experiment had a noise floor of ~10 nA. Note that the noise floor of commercially available Si photodiode is almost 4 orders of magnitude higher than what we can achieve in our FET based MoS_2_ photodetector (noise floor with mean ~3.4 pA and standard deviation of ~4.2 pA). Figure 3j shows the sensitivity of the MoS_2_ photodetector in different detection regimes. The sensitivity (*S*) of a photodetector is defined as the ratio of photocurrent (*I*_PH_) to the luminous flux (*L*_*ϕ*_). The rated sensitivity of the Si photodiode (*S* = 9 nA/lx) and its photoresponse to different *V*_LED_ were used for calibrating *L*_*ϕ*_, which were subsequently used to calculate the sensitivity of the MoS_2_ photodetector in different detection regimes. As shown in Fig. 3j, while the sensitivity of the MoS_2_ photodetector exceeds the sensitivity of the Si photodiode in the ON-state, signal detection is comparatively easier using the Si photodiode owing to its low dark current. Similarly, the sensitivity of the MoS_2_ photodetector is significantly smaller than the Si photodiode in the subthreshold and OFF-state, but signal detection is still possible using the MoS_2_ photodetector owing to orders of magnitude lower dark current. Finally, Fig. 3k shows the comparison of energy expenditure for the Si photodiode and the MoS_2_ photodetector for detection of above-threshold signals. The MoS_2_ photodetector offers orders of magnitude higher energy efficiency when operated in the subthreshold regime, owing to significantly lower dark current. For example, for detecting *V*_LED_ = 2.8 V, the energy consumed by the MoS_2_ photodetector is ~470 pJ at *V*_BG_ = −2 V, compared to 2.8 µJ by the Si photodiode.

### Demonstration of SR in monolayer MoS_2_ photodetector

**Fig. 4:**
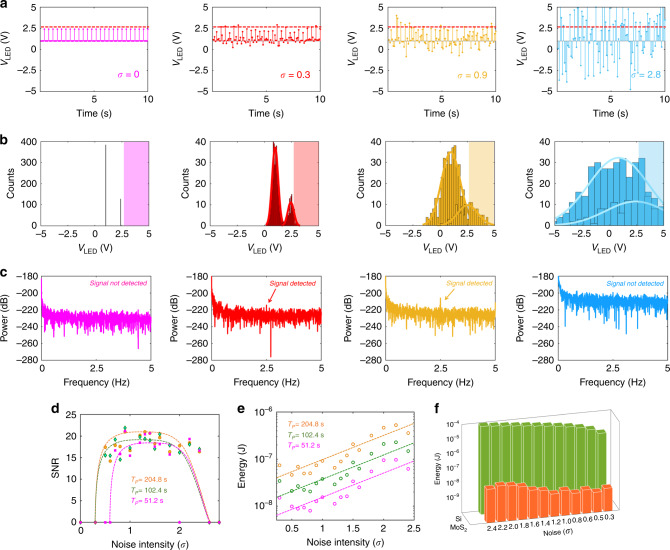
Demonstration of SR in monolayer MoS_2_ photodetector. **a** White Gaussian noise of different standard deviations (*σ*) superimposed on 2.5 Hz periodic and subthreshold LED signal of amplitude *V*_LED_ = 2.4 V and **b** corresponding histogram of the LED voltage distribution. Each histogram shows two Gaussian curves of the same variance but different means, one centered at *V*_LED_ = 1 V (LED OFF) and the other one centered at *V*_LED_ = 2.4 V (Dim LED), marking the level difference between the two states of the LED signal. **c** PSD plots obtained from the FFT of the output current (*I*_DS_) measured in the monolayer MoS_2_ photodetector for a total duration of *T*_P_ = 204.8 s in response to the LED signals in (**a**). The MoS_2_ photodetector was biased in the OFF-state (*V*_BG_ = −2 V). In the presence of a finite and appropriate amount of noise, the subthreshold LED signal is detected. (see Supplementary video files 1–3 for real-time observation of SR in the MoS_2_ photodetector). **d** SNR as a function of *σ* for different sampling time *T*_P_. For very low variance Gaussian noise (*σ* < 0.2 V), the signal hardly crosses the detection threshold of the MoS_2_ photodetector, i.e., *V*_LED_ = 2.6 V. As *σ* increases, a larger fraction of the LED signal corresponding to the 2.4 V level crosses the detection threshold of the MoS_2_ photodetector, increasing the SNR. For even larger variances, the SNR starts to decrease since the tail of the Gaussian distribution centered at *V*_LED_ = 1 V will also start to cross the detection threshold of the MoS_2_ photodetector, obscuring the periodicity of the *V*_LED_ = 2.4 V signal. The SNR curves in this instance show the typical SR response found in biological species such as paddlefish, crayfish, etc. **e** Energy consumption by the MoS_2_ photodetector is plotted as a function of *σ* for different *T*_P_. Since the MoS_2_ photodetector was operated in the deep OFF-state (*V*_BG_ = −2 V) to minimize the dark current, the operating power budget is drastically reduced, making the signal detection extremely energy efficient. For comparison, refer to Supplementary Note 9 for the exploitation  of SR using the Si photodiode (see Supplementary video files 4–6 for real-time observation of SR in the Si photodiode). **f** The energy consumption by the Si photodiode for detecting subthreshold optical signal, exploiting SR, is found to be in the range of a few micro-Joules, which is 1000X higher compared to the MoS_2_ photodetector for sampling time *T*_P_ = 100 s.

In this section, we will seek to extend the detection limit for the MoS_2_ photodetector by exploiting SR. Figure 4a shows a 2.5 Hz periodic and subthreshold LED signal of amplitude *V*_LED_ = 2.4 V with various amount of Gaussian noise added to it. Figure 4b shows the corresponding histogram of the LED voltage distribution. Note that each histogram shows two Gaussian curves of the same variance but different means, i.e. one centered at *V*_LED_ = 1 V (LED OFF) and the other one centered at *V*_LED_ = 2.4 V (Dim LED), marking the level difference between the two states of the LED signal. Figure 4c shows the corresponding PSD obtained using the FFT of the output current (*I*_DS_) response from the MoS_2_ photodetector biased in the OFF-state (*V*_BG_ = −2 V). The current sampling was done at 10 Hz for ~204.8 s. Clearly, in the presence of a finite and appropriate amount of noise, the subthreshold LED signal is detected.

Supplementary video file 1 shows the real-time recording of the blue LED subjected to a 2.5 Hz periodic signal of amplitude *V*_LED_ = 2.4 V and the corresponding PSD of *I*_DS_. No peak appears at 2.5 Hz in the PSD, indicating that the photodetector is unable to detect the LED signal. Supplementary video file 2 shows the real-time recording of the blue LED subjected to a  random signal with Gaussian noise of standard deviation 0.3 V added to a constant LED signal of *V*_LED_ = 2.4 V and corresponding PSD of *I*_DS_. Even in this case, no peak is observed in the PSD, which is consistent with the random nature of the LED signal. These two videos ensure that neither the weak periodic LED signal nor the noisy LED signal can generate an identifiable response in the MoS_2_ photodetector. However, Supplementary video file 3 shows that when random Gaussian noise of standard deviation 0.3 V is added to the 2.5 Hz periodic LED signal of amplitude *V*_LED_ = 2.4 V, the PSD of *I*_DS_ starts to show a distinguishable peak at 2.5 Hz, the strength of which increases as the sampling continues. These videos provide direct evidence of SR in the MoS_2_ photodetector, or, in other words, the constructive role of noise in the detection of the weak periodic signals.

Figure 4d shows the SNR as a function of the variance of the Gaussian noise added to the 2.5 Hz LED signal. For very low variance Gaussian noise (*σ* < 0.2 V), the signal hardly crosses the detection threshold of the MoS_2_ photodetector, i.e., *V*_LED_ = 2.6 V, as can be seen from the histogram in Fig. 4b. As the variance of the noise increases, a larger fraction of the LED signal corresponding to the 2.4 V level crosses the detection threshold of the MoS_2_ photodetector, enhancing the SNR. For even larger variances, the SNR starts to decrease since the tail of the Gaussian distribution centered at *V*_LED_ = 1 V will also start to cross the detection threshold of the MoS_2_ photodetector, obscuring the periodicity of the *V*_LED_ = 2.4 V signal. The SNR curves show the typical SR response found in biological species such as paddlefish, crayfish, etc. Note that irrespective of the nature of the periodic signal and its background, the SNR can be increased by increasing the total sampling time (*T*_P_) for the appropriate noise variance. Finally, Fig. 4e shows the energy consumption by the device for detecting the weak periodic LED signal. Since the MoS_2_ photodetector was operated in the deep OFF-state (*V*_BG_ = −2 V) to minimize the dark current, the operating power budget is drastically reduced, making the signal detection extremely energy efficient. In the present case, the energy dissipation was as frugal as few nano-Joules.

In Supplementary Note 9, we exploit SR for extending the detection limit of a commercially available Si photodiode. Supplementary video file 4 shows the real-time recording of the blue LED subjected to a 2.5 Hz periodic signal of amplitude *V*_LED_ = 2.4 V and the corresponding PSD of the reverse bias current (*I*_PD_) measured in the Si photodiode, which is placed at ~1 cm from the LED. No peak appears at 2.5 Hz in the PSD, indicating that the Si photodiode is unable to detect the LED signal. Supplementary video file 5 shows the real-time recording of the blue LED subjected to a random signal with Gaussian noise of standard deviation 0.4 V added to a constant LED signal of *V*_LED_ = 2.4 V and corresponding PSD of *I*_PD_. Even in this case, no peak is observed in the PSD, which is consistent with the random nature of the LED signal. These two videos ensure that neither the weak periodic LED signal nor the noisy LED signal can generate an identifiable response in the Si photodiode. However, Supplementary video file 6 shows that when random Gaussian noise of standard deviation 0.4 V is added to the 2.5 Hz periodic LED signal of amplitude *V*_LED_ = 2.4 V, the PSD of *I*_DS_ starts to show a distinguishable peak at 2.5 Hz, the strength of which increases as the sampling continues. These videos provide direct evidence of SR in the Si photodiode. The energy consumption by the Si photodiode for detecting subthreshold optical signal, exploiting SR, is found to be in the range of a few micro-Joules, which is ~1000× higher compared to that of the MoS_2_ photodetector. This is due to the fact that the reverse dark current in Si photodiode is orders of magnitude higher than the subthreshold conduction current of the MoS_2_ FET.

Note that in the above experimental demonstration of SR in the MoS_2_ photodetector, the white Gaussian noise was added to the original LED driving voltage to bring the signal randomly above the detection limit of the MoS_2_ photodetector. Therefore, it may appear that the noise has to be in the nature of the signal, which may restrict the use of SR in many real-life applications. This is not true. Supplementary Note 10 shows that noise can be added externally to aid the subthreshold signal detection. The experimental setup consisted of the MoS_2_ photodetector and two blue LEDs, one of which is used as the source of the weak periodic signal (referred to as the “Signal” LED) and the other one as the source of the white Gaussian noise (referred to as the “Noisy” LED). Supplementary video file 7 shows the real-time recording of a 2.5 Hz periodic signal alternating between *V*_LED_ = 1.0 V and *V*_LED_ = 2.6 V applied to the “Signal” LED and the corresponding PSD of *I*_DS_. No peak appears at 2.5 Hz in the PSD, indicating that the MoS_2_ photodetector is unable to detect the LED signal. Supplementary video file 8 shows the real-time recording of the “Noisy” LED subjected to white Gaussian noise of standard deviation 0.1 V and mean of *V*_LED_ = 2.5 V and the corresponding PSD of *I*_DS_. As expected, no peak is observed in the PSD due to the random nature of the “Noisy” LED signal. Interestingly, Supplementary video file 9 shows that a peak appears at 2.5 Hz when the “Signal” LED operates in the presence of the “Noisy” LED. Clearly, the weak periodic signal from the “Signal” LED is detected by the MoS_2_ photodetector only in the presence of the “Noisy” LED and the enhancement in the signal to noise ratio (SNR) increases with increasing sampling time. This experiment conclusively proves that the noise does not need to be in the source of the signal and can be added and adjusted using a separate source that can be integrated with the photodetector. This certainly comes with an energy overhead, although miniscule, in the range of several hundreds of nano-Joules, since the “Noisy” LED must be powered separately. Nevertheless, the above demonstration expands the scope of SR and makes it even more appealing for numerous real-life applications.

## Discussion

While SR has shown evolutionary success in various natural sensors, ensuring the survival of species in resource-constrained environments, its benefits have remained largely underexplored in solid-state sensors. This is not surprising since conventional approaches have been widely successful although power hungry and resource intensive. However, with the emergence of newer technologies, such as the IoT, that demand energy-efficient sensors, deployable in remote locations without continuous source of electrical energy, the time has come to rekindle the concept of SR. Here we have demonstrated how SR can be exploited in monolayer MoS_2_ based photodetector to detect ultra-low intensity optical signals from a distant LED, which  are otherwise below the detection threshold. We have also demonstrated the significant energy benefits of utilizing SR for photodetection. We reemphasize that SR does not need to be a standalone technology. Instead, as we have shown, SR can be used to extend the detection limit of existing state-of-the-art sensors, such as commercially available Si photodiodes. We have also demonstrated that the noise does not need to be in the nature of the signal and can be tuned and added externally. Overall, our approach differs from conventional thinking since we harness noise instead of suppressing it for detecting subthreshold signals. This can mark a paradigm shift in future high precision and ultra-low-power sensor design. Our approach is simple and generic, and can be extended to practically any sensors used in commercial or defense-related applications, where energy and resources are not abundant.

## Methods

### Synthesis of monolayer MoS_2_

Monolayer MoS_2_ was deposited on epi-ready 2” c-sapphire substrate by metalorganic chemical vapor deposition (MOCVD). An inductively heated graphite susceptor equipped with wafer rotation in a cold-wall horizontal reactor was used to achieve uniform monolayer deposition (schematic in Fig. 1a) as previously described^54^. Molybdenum hexacarbonyl (Mo(CO)_6_) and hydrogen sulfide (H_2_S) were used as precursors. Mo(CO)_6_, maintained at 10 °C and 950 Torr in a stainless steel bubbler, was used to deliver 0.036 sccm of the metal precursor for the growth, while 400 sccm of H_2_S was used for the process. MoS_2_ deposition was carried out at 1000 °C and 50 Torr in H_2_ ambient, where monolayer growth was achieved in 18 min. The substrate was first heated to 1000 °C in H_2_ and maintained for 10 min before the growth was initiated. After growth, the substrate was cooled in H_2_S to 300 °C to inhibit decomposition of the MoS_2_ films.

### Characterization of monolayer MoS_2_

Surface coverage and thickness were measured using peak-force tapping mode in a Bruker Icon AFM using scanasyst AFM tips with a nominal tip radius of ∼2 nm and a spring constant of 0.4 N/m. Raman and PL measurements were performed using a HORIBA LabRAM HR Evolution Raman microscope with laser wavelengths of 532 nm. Raman spectra were collected with 1800 grooves per mm grating, while PL measurements were conducted with 300 grooves per mm grating. The Raman and PL maps were acquired over a 5 × 5 µm^2^ area. X-ray diffraction characterization of MoS_2_ films was carried out with a PANalytical MRD diffractometer with a 5-axis cradle. X-rays were generated in a standard Cu anode X-ray tube operated at 40 kV accelerating voltage and 45 mA filament current. Cu K line was filtered by a mirror with 1/4° slit and Ni filter on the primary beam side. On the diffracted beam side, an 0.27° parallel plate collimator with 0.04 rad Soller slits with PIXcell detector in open detector mode was employed. Samples' surface was ~2–4° away from the X-ray incidence plane such that diffraction caused by the (h k i 0) planes, to determine the in-plane epitaxial relation of the film with respect to a substrate, could be measured^35^.

### Transfer of monolayer MoS_2_

To fabricate the MoS_2_ FETs, MoS_2_ film grown on sapphire was transferred onto alumina substrate using PMMA (polymethyl-methacrylate) assisted wet transfer process. MoS_2_ on sapphire substrate was spin coated with PMMA and then baked at 180 °C for 90 s. The corners of the spin-coated film were scratched using a razor blade and immersed inside 1 M NaOH solution kept at 90 °C. Capillary action causes the  NaOH to be drawn into the substrate/film interface, separating the PMMA/MoS_2_ film from the sapphire substrate. The separated film was rinsed multiple times inside a water bath and finally transferred onto the 50 nm alumina substrate and then baked at 50 °C and 70 °C for 10 min each to remove moisture and residual PMMA, ensuring a pristine interface.

### Gate dielectric fabrication

Direct replacement of thermally oxidized SiO_2_ with a high-κ dielectric such as Al_2_O_3_ grown via ALD is a logical choice to scale the effective oxide thickness (EOT). However, we found that Al_2_O_3_/p^++^-Si interface is not ideal for back-gated FET fabrication owing to higher gate leakage current, more interface trap-states, and large hysteresis, which negatively impact the performance of the device. Replacing Si with Pt, a large work function metal (5.6 eV), allows minimal hysteresis and trap state effects^55^. Since Pt readily forms a Pt silicide at temperatures as low as 300 °C, a 20 nm TiN diffusion barrier deposited by reactive sputtering was placed between the p^++^ Si and the Pt, permitting subsequent high-temperature processing^56^. This conductive TiN diffusion barrier allows the back-gate voltage to be applied to the substrate, thus simplifying the fabrication and measurement procedures. The polycrystalline Pt introduces very little surface roughness to the final Al_2_O_3_ surface, with a rms roughness of 0.7 nm.

### Device fabrication

Back-gated FETs were fabricated using e-beam lithography. To define the channel region, the substrate was spin-coated with PMMA and baked at 180 °C for 90 s. The photoresist was then exposed to e-beam and developed using 1:1 mixture of 4-methyl-2-pentanone (MIBK) and 2 propanols (IPA). The monolayer MoS_2_ film was subsequently etched using sulfur hexafluoride (SF_6_) at 5 °C for 30 s. Next, the sample was rinsed in acetone and IPA to remove the photoresist. In order to fabricate the source/drain contacts, the substrate was again spin-coated with MMA and PMMA, followed by the e-beam lithography, developing using MIBK and IPA, and e-beam evaporation of a 40 nm Ni/30 nm Au stack. Finally, the photoresist was rinsed away by lift-off process using acetone and IPA.

### Electrical measurements

Electrical measurements were performed in air inside a Lakeshore probe station using a B1500A Keysight semiconductor parameter analyzer.

## Supplementary information

Supplementary Information

Peer Review File

Descriptions of Additional Supplementary Files

Supplementary_Video_File_1

Supplementary_Video_File_2

Supplementary_Video_File_3

Supplementary_Video_File_4

Supplementary_Video_File_5

Supplementary_Video_File_6

Supplementary_Video_File_7

Supplementary_Video_File_8

Supplementary_Video_File_9

## Data Availability

The datasets generated during and/or analyzed during the current study are available from the corresponding author on reasonable request.

## Code availability

The codes used for plotting the data are available from the corresponding authors on reasonable request.
