# Supplementary material for: Stochastic resonance in MoS2 photodetector
Source: Nat Commun. 2020 Sep 2;11:4406. doi: 10.1038/s41467-020-18195-0 (PMC7468142; doi:10.1038/s41467-020-18195-0)
Supplement: Supplementary file 1 — Supplementary Information [file 41467_2020_18195_MOESM1_ESM.pdf]

Supplementary Information

Stochastic Resonance in MoS_2 Photodetector

Akhil Dodda[1], Aaryan Oberoi[1], Amritanand Sebastian[1], Tanushree H Choudhury[2], Joan M Redwing[2,3] & Saptarshi Das[1,2,3,*]

[1]*Department of Engineering Science and Mechanics, Pennsylvania State University, University Park, PA, 16802, USA*

[2]*Department of Materials Science and Engineering, Pennsylvania State University, University Park, PA, 16802, USA*

[4]*Materials Research Institute, Pennsylvania State University, University Park, PA 16802, USA*

These Authors Contributed Equally: Akhil Dodda, Aaryan Oberoi.

Corresponding Author: Saptarshi Das

Supplementary Note 1:

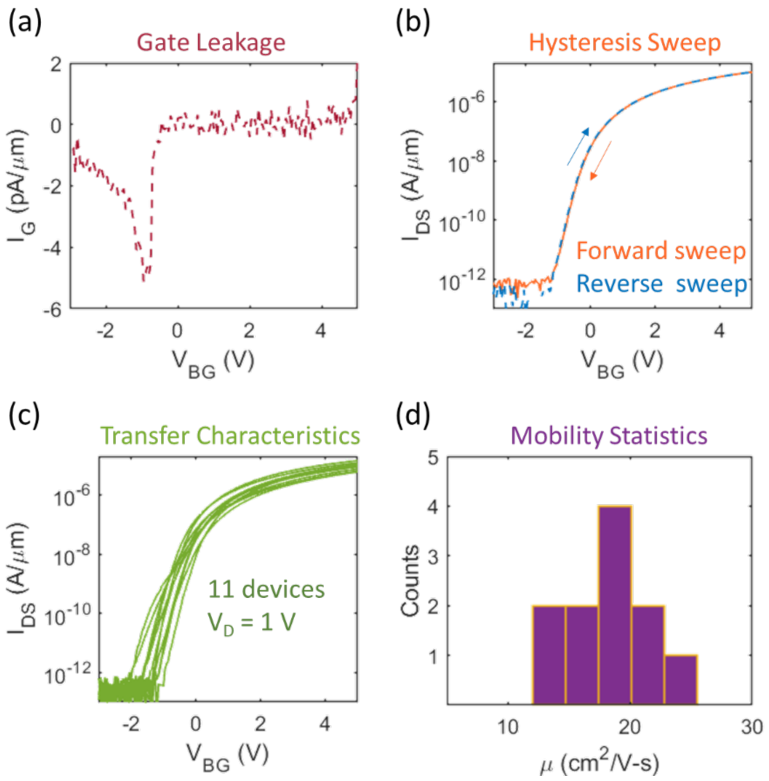

Supplementary Figure 1. Gate leakage and hysteresis characteristics of MoS_2 FET and device to device variation across the wafer. *a) Gate leakage I_G as a function of back-gate bias (V_{BG}). b) Device characteristics showing forward and reverse V_{BG} sweeps from 5 V to -3 V. No hysteresis is observed in the device characteristics indicating relatively clean interface between the monolayer MoS_2 and the gate dielectric stack. Note that the lack of hysteresis in the transfer characteristics is not an indication of lack of charge trapping at the interface. In fact, the deviation of the subthreshold slope (SS) of the MoS_2 FET from its ideal value of 60 mV/decade, is a clear evidence of trapped charges on the surface of the MoS_2, in the gate dielectric, at the MoS_2/dielectric interface, or in the MoS_2 itself [1-3]. The SS is given by the formula: $SS = \frac{k_B T}{q} ln10\left(1+\frac{C_D+C_{IT}}{C_{ox}}\right) = 60\left(1+\frac{C_D+C_{IT}}{C_{ox}}\right)\ mV/decade$, where, k_B is the Boltzmann constant, q is the electronic charge, T is the temperature and C_D, C_{IT} and C_{ox} are respectively, the depletion capacitance, interface trap capacitance and oxide capacitance. For an ultra-thin-body, fully depleted, semiconducting, channel material such as monolayer MoS_2, C_D is negligible and hence the deviation in SS from its ideal value of 60 mV/decade at room temperature can be attributed to the presence of interface trap states, which is expected given the low technology level of MoS_2 devices. c) Transfer characteristics for 11 representative monolayer MoS_2 FETs measured across the entire substrate show minimal device to device variation indicating high quality MOCVD growth over a large area. d) Histogram of field effect mobility (μ_{FE}) extracted from the peak transconductance ($\mu_{FE} = \frac{\partial I_{DS}}{\partial V_{BG}} \times \frac{L}{C_{ox}*W*V_D}$) corresponding to the 11 devices. L is the channel length, W is the channel width, C_{ox} is the oxide capacitance and V_D is the drain bias.*

Supplementary Note 2.

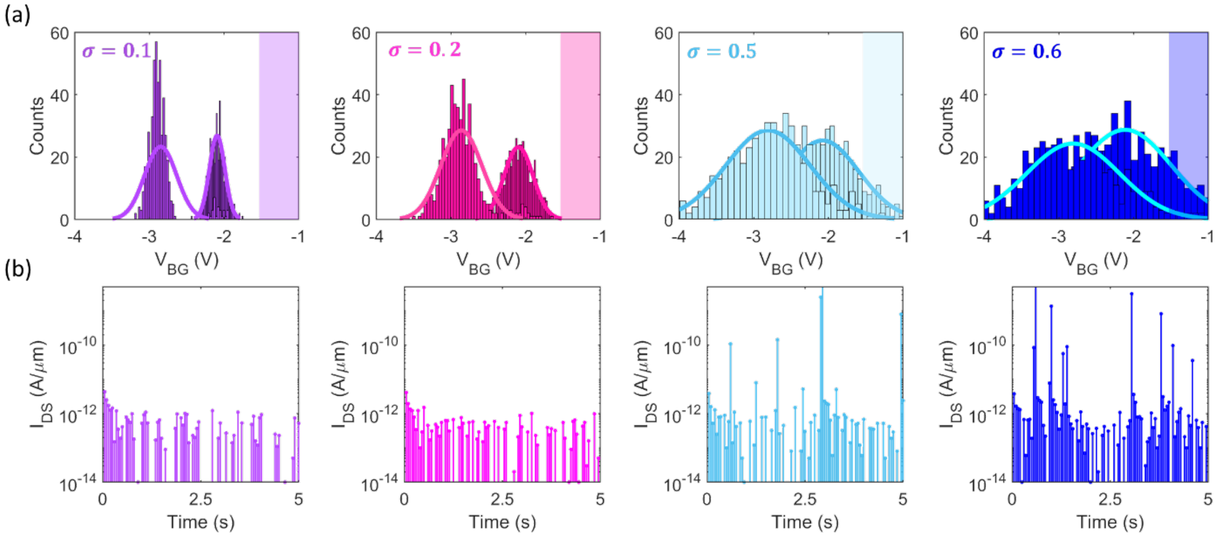

Supplementary Figure 2. Histogram of the back-gate voltage distribution and corresponding output current response in MoS_2 FET. *a) Histogram of the voltage distribution applied to the back-gate of the monolayer MoS_2 FET from Fig. 2 with different standard deviation of noise (σ). The shaded region represents the operation of the device above threshold i.e. $V_{BG} > -1.5$ V. For very low variance Gaussian noise (σ = 0.2 V), none of the two signal levels crosses the detection threshold (V_{BG} = -1.5 V), whereas, for very high variance Gaussian noise (σ = 0.6 V), both signal levels cross the detection threshold and obscures the periodicity of the signal. However, for appropriate noise variance mostly one of the two signal levels cross the detection threshold allowing the signal frequency to be detected. b) Output current I_{DS} measured from the MoS_2 FET for various σ.*

Supplementary Note 3.

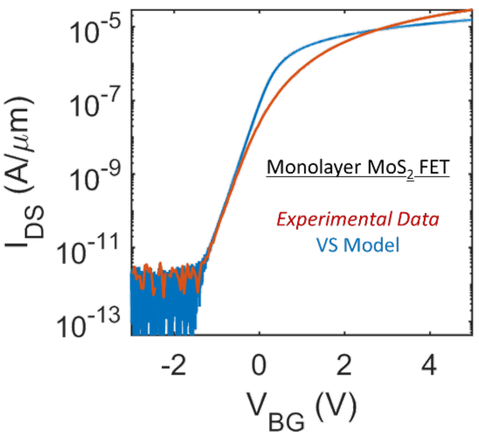

Supplementary Figure 3. Virtual source (VS) model for fitting experimental monolayer MoS_2 FET characteristics. *Experimental and simulated transfer characteristics of MoS_2 FET using the Virtual Source (VS) model [4]. In the VS model, both the subthreshold and the above threshold behavior is captured through a single semi-empirical and phenomenological relationship that describes the transition in channel charge (Q_{CH}) from weak to strong inversion.*

$$Q_{CH} = C_{BG} m \frac{k_B T}{q} log\left[1 + exp\left(\frac{V_{BG} - V_T}{mk_B T/q}\right)\right];\ R_{CH} = \frac{L_{CH}}{\mu_N Q_{CH}};\ I_{DS} = \frac{V_{DS}}{R_{CH}}$$

In the above equation, C_{BG} =1.78x10^{-3} F/m^2, is the back-gate capacitance of 50 nm Al_2O_3 with relative dielectric constant of ~9.1 [5]. The band movement factor, $m = \left(1 + \frac{C_D + C_{IT}}{C_{ox}}\right)$, was obtained from the subthreshold slope ($SS = mk_B T ln10$) of the experimental transfer characteristics and was found to be 4.9. The threshold voltage V_T was found to be 0.2V. Further, $L_{CH} = 1\ \mu m$ is the channel length, and μ_N is the carrier mobility for electrons in MoS_2 which was extracted from the peak transconductance value and was found to be 16 cm^2/V-s. Note, that in the subthreshold regime, the inversion charge increases exponentially with V_{BG}, whereas above threshold, the inversion charge is a linear function of V_{BG}, which is seamlessly captured through the VS model. The noise floor of the measurement instrument is modeled using a white Gaussian leakage current of mean 3.4 pA and standard deviation of 4.2 pA also extracted from the experimental data.

Supplementary Note 4.

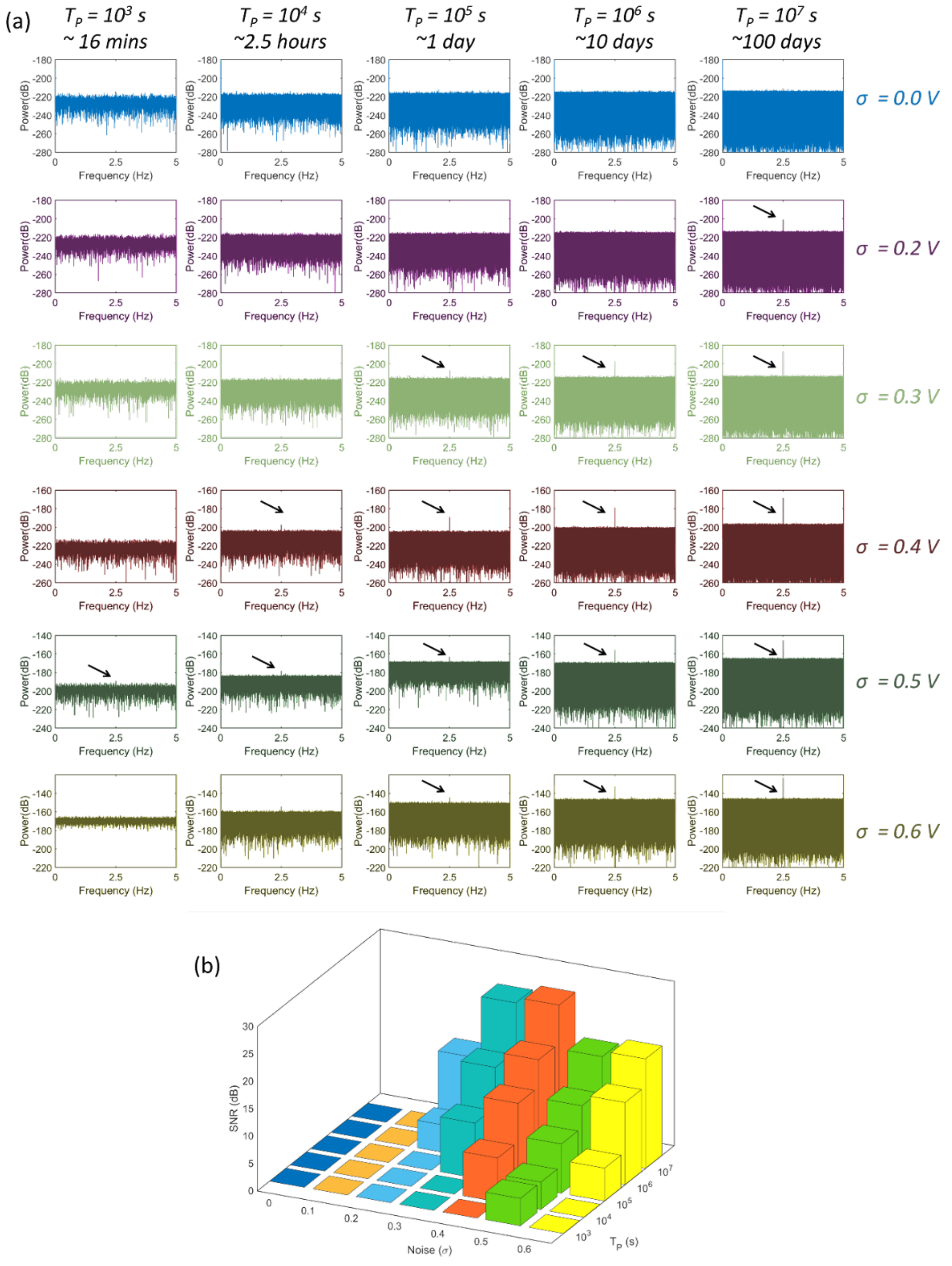

Supplementary Figure 4. Interplay of standard deviation of noise and latency in SR. *a) PSD of I_{DS} in response to a 2.5 Hz periodic input signal of amplitude 0.2 V with Gaussian noise of different standard deviations (σ) applied to the back gate in the OFF-state (V_{BG} = -2.5 V) for an averaging period that ranges from 10^3 s to 10^7 s. b) SNR values extracted using (a). As expected, no peaks are observed for σ = 0 V, i.e. without noise even if the sampling is done for an infinitely long period of time. For small σ = 0.2 V, the peak appears after sampling for 10^7 s, whereas, for optimum σ = 0.5 V, the peak appears after 10^3 s. These simulation results indicate that the SR induced enhancement of SNR can be achieved using any finite value of σ at the expense of increased latency.*

Supplementary Note 5:

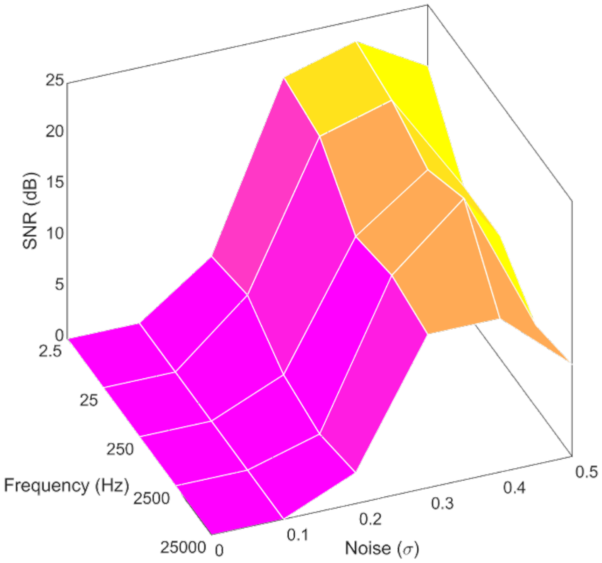

Supplementary Figure 5. Signal frequency and SR. *The surface plot shows the simulation results for the signal to noise ratio (SNR) extracted from the PSD of* I_{DS} *in response to periodic input signal of amplitude 0.2 V of different frequencies applied to the back gate of* MoS_2 *FET in the OFF-state (*V_{BG} = *-2.5 V) with Gaussian noise of different standard deviations (σ). The sampling frequency was 100 kHz and total sampling time was 100 s. Clearly, the characteristic SR traces show minimal change as a function of the signal frequency. In fact, some earlier work has explored THz wave detection by GaAs nanowire based FETs using SR [6].*

Supplementary Note 6:

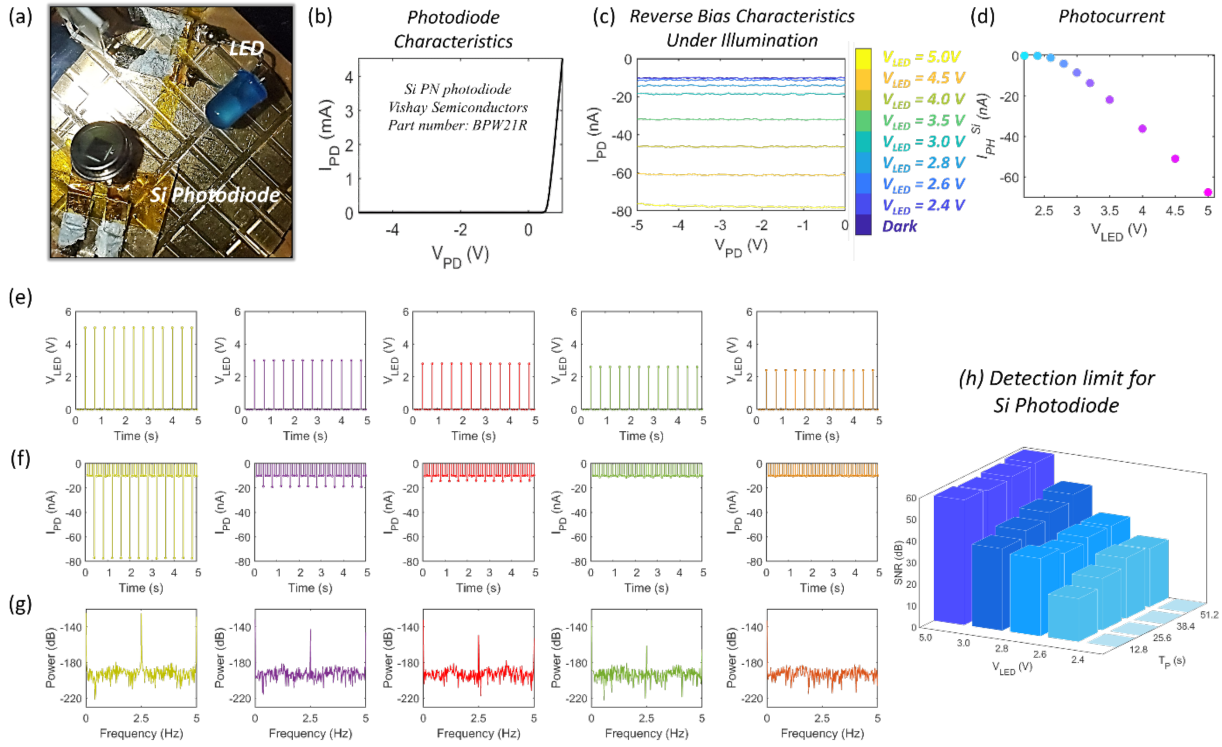

Supplementary Figure 6. Detection limit for commercial Si photodiode. *a) Experimental set-up showing a Si PN photodiode purchased from Vishay Semiconductors, part number: BPW21R placed at ~1 cm from a blue LED. b) current (I_{PD}) versus voltage (V_{PD}) characteristics of the Si PN photodiode measured in dark. c) Reverse bias diode characteristics under different illuminations from the blue LED. d) Photocurrent ($I_{PH}{}^{Si}$) as a function of V_{LED}. The threshold for detection is V_{LED} = 2.4 V, below which the dark current in the Si photodiode dominates, limiting the detection of low-intensity blue light from the distant LED. The rated reverse dark current for the Si photodiode was found to be 2 nA (typical), and 30 nA (maximum). The Si photodiode used in our experiment had a noise floor of ~10 nA. e) 2.5 Hz periodic LED signals of different amplitudes. f) Corresponding reverse current (I_{PD}) in Si photodiode biased at V_R = -5 V. Current sampling was done at 10 Hz for ~ 51.2 s. g) Corresponding power spectral density (PSD) obtained using the fast Fourier transform (FFT) of I_{PD}. h) The signal to noise ratio (SNR) extracted from the PSD. The SNR decreases monotonically with the decreasing LED intensity and eventually vanishes as the photo generated current drops below the dark current at V_{LED} = 2.4 V. The SNR increases with the total sampling time only when the signal is above the detection threshold.*

Supplementary Note 7:

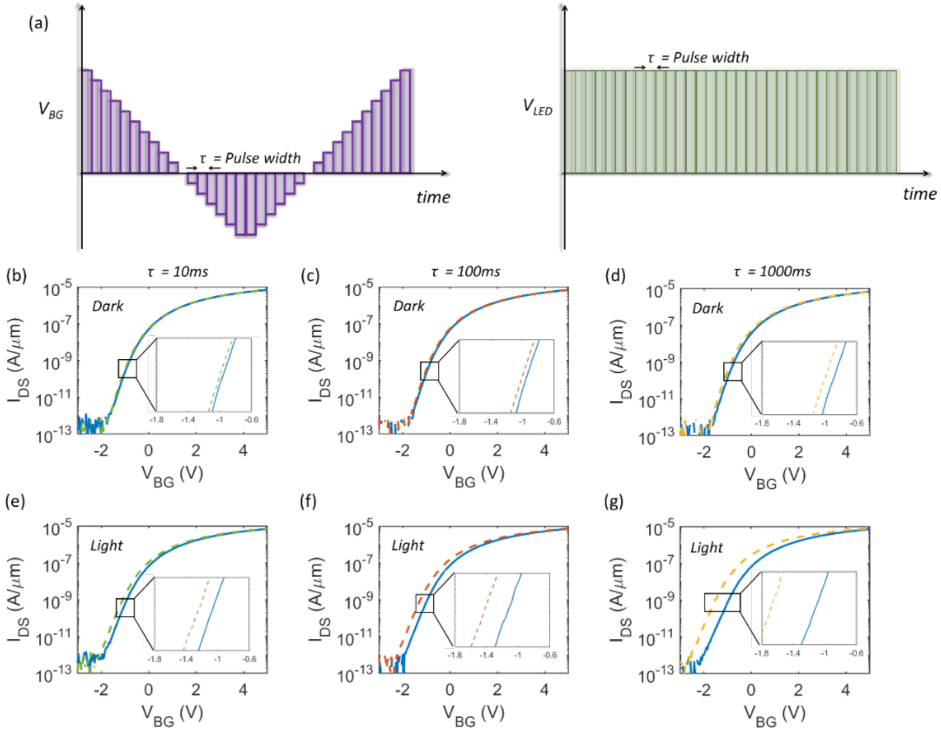

(h) *Hysteresis Window (V_{HW})*

Pulse width / *Illumination*	*10ms*	*100ms*	*1000ms*
Dark (V_{LED} = 0 V)	*40mV*	*60mV*	*120mV*
Light (V_{LED} = 5 V)	*180mV*	*300mV*	*590mV*

Supplementary Figure 7. Sweep rate dependent hysteresis in MoS2 FET. *a) Measurement procedure where both the back-gate voltage (V_{BG}) and LED voltage (V_{LED}) are pulsed simultaneously. Hysteresis characteristics of MoS_2 FET under dark for b) τ = 10ms, c) τ = 100ms, d) τ = 1000ms and under illumination from the blue LED glowing at its full intensity at V_{LED} = 5.0 V for e) τ = 10ms, f) τ = 100ms, g) τ = 1000ms . h) Table showing the extracted hysteresis window (ΔV_{HW}) using the insets of (b)-(g). The sweep rate dependent hysteresis can be explained from the fact that MoS_2 devices are dominated by donor-like trap states which follow Fermi−Dirac distribution with the Fermi level being determined by the equilibrium Fermi level of the MoS_2 channel [2, 3, 7]. Under negative V_{BG} the Fermi level in the monolayer MoS_2 channel moves closer to the valence band. This removes electrons from the trap states above the Fermi level making them positively charged and causing a negative shift in the threshold voltage (V_{TH}). Under positive V_{BG}, electrons refill the traps, rendering them neutral and leading to a positive V_{TH} shift. Electrons remain trapped until the reverse sweep, during which they de-trap [8]. Smaller pulse widths or faster sweep rates prevent traps from becoming populated [9] or in case of water dipoles restrict them from becoming aligned with the gate-induced field [7]. The increase in hysteresis window under illumination for any given sweep rate is expected since electron-photon interaction promotes trapping and causes further negative shift in the threshold voltage (V_{TH}).*

Supplementary Note 8:

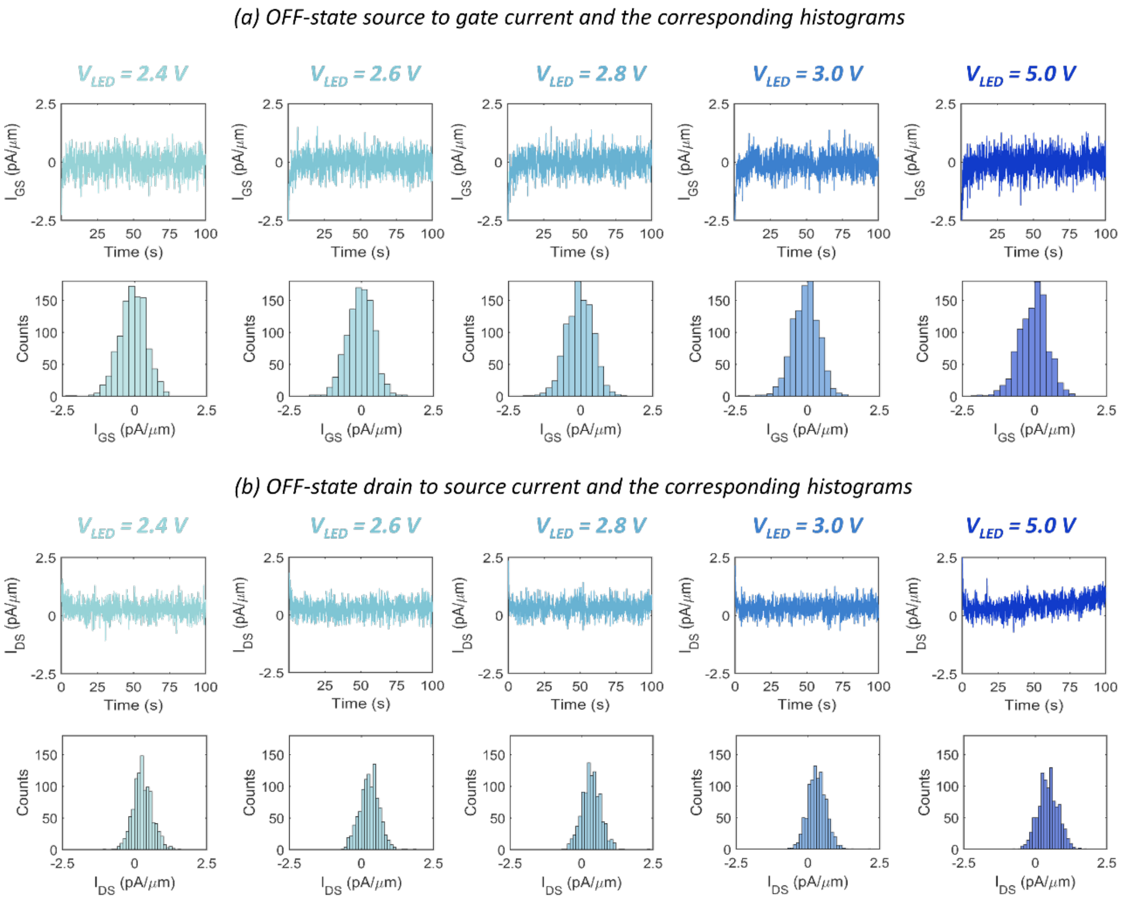

Supplementary Figure 8. Leakage current in MoS2 FET under illumination. *a) Source to gate current (I_{GS}) and b) source to drain current (I_{DS}) sampled at 10 Hz in the OFF state (V_{BG} = - 2.5 V) under different illumination conditions along with their corresponding histograms of current distribution. In all instances, the current appears to be fluctuating randomly following a zero mean Gaussian distribution. The standard deviations show none to very minimal change between different illuminations. Therefore, we can rule out light enhanced gate leakage as the phototransduction mechanism in MoS_2 photodetector.*

Supplementary Note 9:

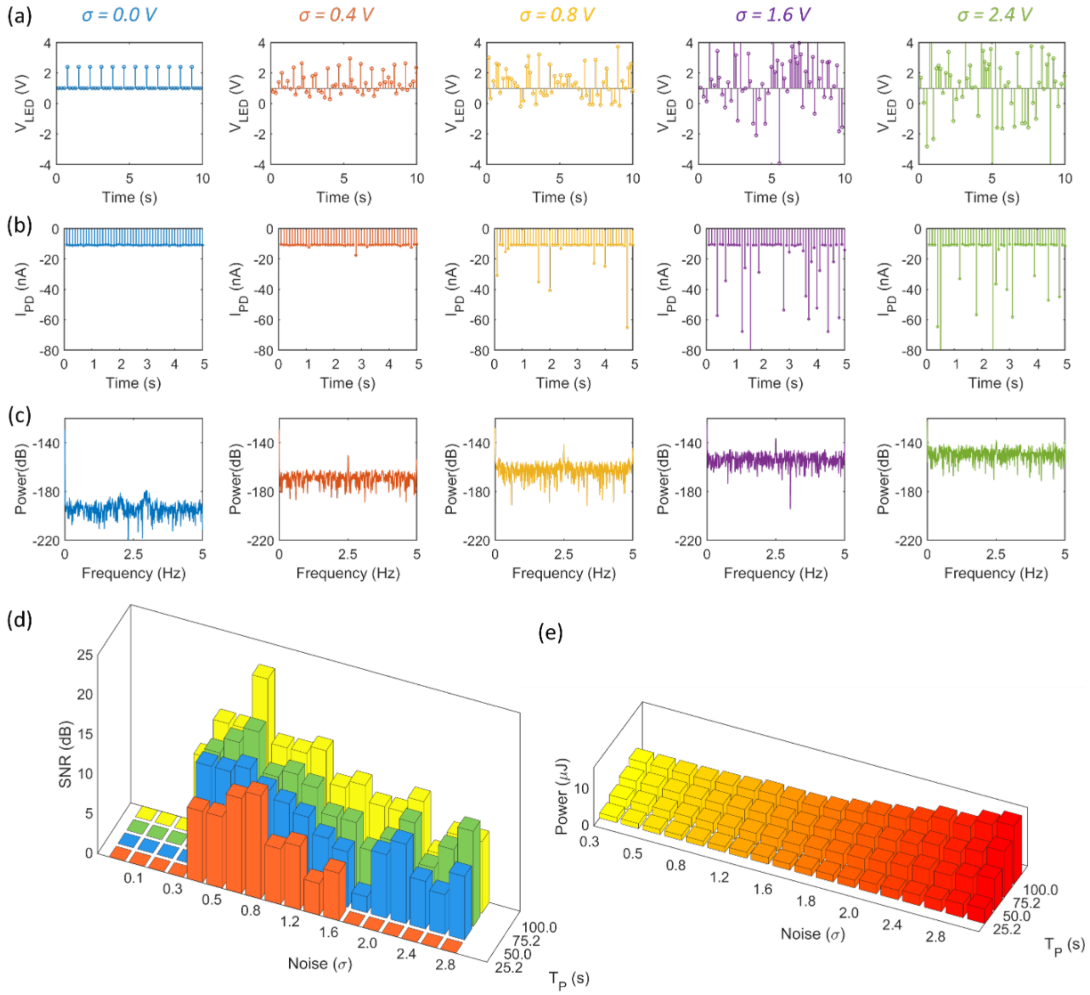

Supplementary Figure 9. Stochastic resonance in Si photodiode. *a) 2.5 Hz periodic LED signals alternating between V_{LED} = 1.0 V and V_{LED} = 2.4 V with various amount of Gaussian noise added to it. b) Corresponding reverse bias current (I_{PD}) in Si photodetector biased at V_R = -5 V, and c) the PSD obtained using FFT of I_{PD}. Current sampling was done at 10 Hz for ~ 100 s. Clearly, in the presence of finite and appropriate amount of noise the subthreshold LED signal is detected. d) The signal to noise ratio (SNR) as a function of the variance of the Gaussian noise shows SR in Si photodiode. The SNR can be increased by increasing the total the sampling time (T_P). e) The energy consumption by the Si photodiode for detecting the weak periodic LED signal.* ***Supplementary video file 4*** *shows the real time recording of the blue LED subjected to a 2.5 Hz periodic signal of amplitude V_{LED} = 2.4 V and the corresponding PSD of I_{PD}. No peak appears at 2.5 Hz in the PSD indicating that the Si photodiode is unable to detect the LED signal.* ***Supplementary video file 5*** *shows the real time recording of the blue LED subjected to random signal with Gaussian noise of standard deviation 0.4 V added to a constant LED signal of V_{LED} = 2.4 V and corresponding PSD of I_{PD}. Even in this case, no peak is observed in the PSD, which is consistent with the random nature of the LED signal. These two videos ensure that neither the weak periodic LED signal nor the noisy LED signal can generate identifiable response in the Si photodiode. However,* ***Supplementary video file 6*** *shows that when random Gaussian noise of standard deviation 0.4 V is added to the 2.5 Hz periodic LED signal of amplitude V_{LED} = 2.4 V, the PSD of I_{DS} starts to show a distinguishable peak at 2.5 Hz, whose strength increases as the sampling continues. These videos provide direct evidence of SR in Si photodiode.*

Supplementary Note 10:

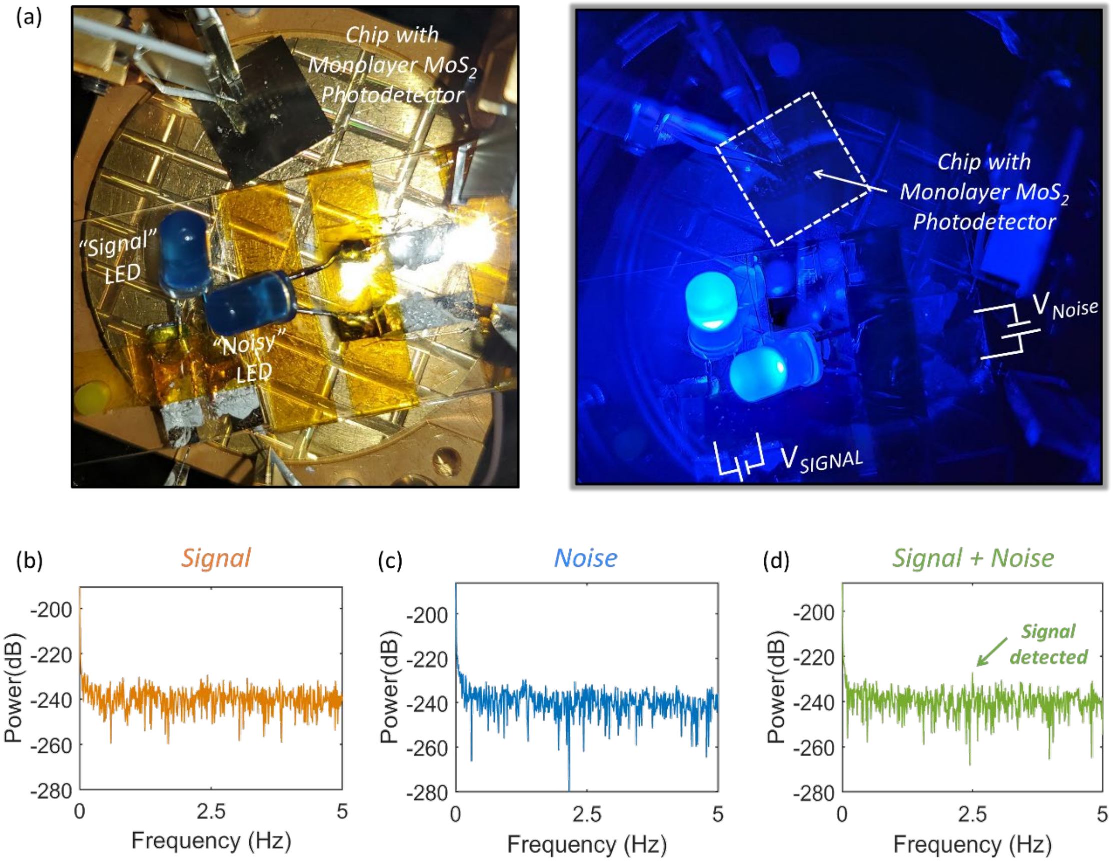

Supplementary Figure 10. Demonstration of SR using external noise source. *a) Experimental setup consisting of the MoS_2 photodetector and two blue LEDs, one of which is used as the source of the weak periodic signal (referred to as the "Signal" LED) and the other one is used as the source of the white Gaussian noise (referred to as the "Noisy" LED). b) Power spectral density (PSD) obtained using the fast Fourier transform (FFT) of the output current (I_{DS}) in the MoS_2 photodetector biased in the OFF state (V_{BG} = -1 V) in response to a 2.5 Hz periodic signal alternating between V_{LED} = 1.0 V and V_{LED} = 2.6 V applied to the "Signal" LED for 100s. No peak appears at 2.5 Hz in the PSD indicating that the photodetector is unable to detect the LED signal. c) PSD of I_{DS} in the MoS_2 photodetector under same biasing conditions in response to white Gaussian noise of standard deviation 0.1 V and mean of V_{LED} = 2.5 V applied to the "Noisy" LED for 100s. No peak is observed in the PSD due to the random nature of the "Noisy" LED signal. d) PSD of I_{DS} in the MoS_2 photodetector when the "Signal" LED operates in the presence of the "Noisy" LED. A distinct peak appears at 2.5 Hz confirm signal detection. This demonstration shows that the noise does not need to be in the nature of the signal and can be added externally for exploiting SR based detection of ultra-low-intensity signal. Current sampling was done at 10 Hz for all instances.*

Supplementary References

[1] I. M. Datye, A. J. Gabourie, C. D. English, K. K. Smithe, C. J. McClellan, N. C. Wang*, et al.*, "Reduction of hysteresis in MoS2 transistors using pulsed voltage measurements," *2D Materials,* vol. 6, p. 011004, 2018.

[2] A. J. Arnold, A. Razavieh, J. R. Nasr, D. S. Schulman, C. M. Eichfeld, and S. Das, "Mimicking Neurotransmitter Release in Chemical Synapses via Hysteresis Engineering in MoS2 Transistors," *ACS nano,* vol. 11, pp. 3110-3118, 2017.

[3] D. J. Late, B. Liu, H. S. Matte, V. P. Dravid, and C. N. Rao, "Hysteresis in single-layer MoS2 field effect transistors," *ACS Nano,* vol. 6, pp. 5635-41, Jun 26 2012.

[4] M. S. Lundstrom and D. A. Antoniadis, "Compact models and the physics of nanoscale FETs," *IEEE Transactions on Electron Devices,* vol. 61, pp. 225-233, 2013.

[5] A. Sebastian, F. Zhang, A. Dodda, D. May-Rawding, H. Liu, T. Zhang*, et al.*, "Electrochemical Polishing of Two-Dimensional Materials," *ACS Nano,* vol. 13, pp. 78-86, 2019/01/22 2019.

[6] S. Kasai, Y. Shiratori, K. Miura, H. Shibata, Y. Nakano, and T. Muramatsu, "THz wave detection by gate-controlled GaAs nanowire devices," in *OECC 2010 Technical Digest*, 2010, pp. 530-531.

[7] A. Di Bartolomeo, L. Genovese, F. Giubileo, L. Iemmo, G. Luongo, T. Foller*, et al.*, "Hysteresis in the transfer characteristics of MoS2 transistors," *2D Materials,* vol. 5, p. 015014, 2017.

[8] F. Heiman and G. Warfield, "The effects of oxide traps on the MOS capacitance," *IEEE Transactions on Electron Devices,* vol. 12, pp. 167-178, 1965.

[9] D. Estrada, S. Dutta, A. Liao, and E. Pop, "Reduction of hysteresis for carbon nanotube mobility measurements using pulsed characterization," *Nanotechnology,* vol. 21, p. 085702, 2010.
