# Supplementary material for: Stochastic resonance in MoS2 photodetector
Source: Nat Commun. 2020 Sep 2;11:4406. doi: 10.1038/s41467-020-18195-0 (PMC7468142; doi:10.1038/s41467-020-18195-0)
Supplement: Supplementary file 3 — Descriptions of Additional Supplementary Files [file 41467_2020_18195_MOESM3_ESM.pdf]

Descriptions of Additional Supplementary Files

Supplementary Video 1

Description: SR in MoS2 Photodetector Weak_Periodic_LED_Signal: Real time recording of the blue LED subjected to a 2.5 Hz periodic signal of amplitude V_LED = 2.4 V and the corresponding PSD of I_DS. No peak appears at 2.5 Hz in the PSD indicating that the photodetector is unable to detect the LED.

Supplementary Video 2

Description: SR in MoS2 Photodetector Random_LED_Signal: Real time recording of the blue LED subjected to random signal with Gaussian noise of standard deviation 0.3 V added to a constant LED signal of V_LED = 2.4 V and corresponding PSD of I_DS. Even in this case, no peak is observed in the PSD, which is consistent with the random nature of the LED signal.

Supplementary Video 3

Description: SR in MoS2 Photodetector Weak_Periodic_LED_Signal_with_Optimum_Noise: Real time recording of random Gaussian noise of standard deviation 0.3 V is added to the 2.5 Hz periodic LED signal of amplitude V_LED = 2.4 V, the PSD of I_DS starts to show a distinguishable peak at 2.5 Hz, whose strength increases as the sampling continues.

Supplementary Video 4

Description: SR in Si Photodiode Weak_Periodic_LED_Signal: Real time recording of the blue LED subjected to a 2.5 Hz periodic signal of amplitude V_LED = 2.4 V and the corresponding PSD of reverse bias current (I_PD) measured in the Si photodiode. No peak appears at 2.5 Hz in the PSD indicating that the Si photodiode is unable to detect the LED signal.

Supplementary Video 5

Description: SR in Si Photodiode Random_LED_Signal: Real time recording of the blue LED subjected to random signal with Gaussian noise of standard deviation 0.4 V added to a constant LED signal of V_LED = 2.4 V and corresponding PSD of I_PD. Even in this case, no peak is observed in the PSD, which is consistent with the random nature of the LED signal.

Supplementary Video 6

Description: SR in Si Photodiode Weak_Periodic_LED_Signal_with_Optimum_Noise: Real time recording of random Gaussian noise of standard deviation 0.4 V is added to the 2.5 Hz periodic LED signal of amplitude V_LED = 2.4 V, the PSD of I_DS starts to show a distinguishable peak at 2.5 Hz, whose strength increases as the sampling continues. These videos provide direct evidence of SR in Si photodiode.

Supplementary Video 7

Description: SR in MoS2 Photodetector with External Noise Source Signal_LED: Real time recording of a 2.5 Hz periodic signal alternating between V_LED = 1.0 V and V_LED = 2.6 V applied to the "Signal" LED and the corresponding PSD of I_DS. No peak appears at 2.5 Hz in the PSD indicating that the MoS2 photodetector is unable to detect the LED signal.

Supplementary Video 8

Description: SR in MoS2 Photodetector with External Noise Source Noisy_LED: Real time recording of the "Noisy" LED subjected to white Gaussian noise of standard deviation 0.1 V and mean of V_LED = 2.5 V and the corresponding PSD of I_DS. As expected, no peak is observed in the PSD due to the random nature of the "Noisy" LED signal.

Supplementary Video 9

Description: SR in MoS2 Photodetector with External Noise Source Signal_LED_with_Noisy_LED: Real time recording shows that a peak appears at 2.5 Hz when the "Signal" LED operates in the presence of the "Noisy" LED. Clearly, the weak periodic signal from the "Signal" LED is detected by the MoS2 photodetector only in the presence of the "Noisy" LED and the enhancement in the signal to noise ratio (SNR) increases with increasing sampling time. This experiment conclusively proves that the noise does not need to be in the source of the signal and can be added and adjusted using a separate source which can be integrated with the photodetector.
