# Supplementary material for: Stochastic resonance in MoS2 photodetector
Source: Nat Commun. 2020 Sep 2;11:4406. doi: 10.1038/s41467-020-18195-0 (PMC7468142; doi:10.1038/s41467-020-18195-0)
Supplement: Supplementary file 2 — Peer Review File [file 41467_2020_18195_MOESM2_ESM.pdf]

Editorial Note: This manuscript has been previously reviewed at another journal that is not operating a transparent peer review scheme. The manuscript was considered suitable for publication without further review at *Nature Communications.*
